# Adsorption Kinetic Model Predicts and Improves Reliability of Electrochemical Serotonin Detection

**DOI:** 10.3390/mps6010006

**Published:** 2023-01-09

**Authors:** Ashley Augustiny Chapin, Jinjing Han, Reza Ghodssi

**Affiliations:** 1 Fischell Department of Bioengineering, University of Maryland, College Park, MD 20742, USA; 2 Fischell Institute for Biomedical Devices, University of Maryland, College Park, MD 20742, USA; 3 Institute for Systems Research, University of Maryland, College Park, MD 20742, USA; 4 Department of Electrical and Computer Engineering, University of Maryland, College Park, MD 20742, USA

**Keywords:** electrochemical biosensor, adsorption, modeling, neurotransmitter

## Abstract

Serotonin (5-HT) is a neurotransmitter involved in many biophysiological processes in the brain and in the gastrointestinal tract. Electrochemical methods are commonly used to quantify 5-HT, but their reliability may suffer due to the time-dependent nature of adsorption-limited 5-HT detection, as well as electrode fouling over repeated measurements. Mathematical characterization and modeling of adsorption-based electrochemical signal generation would improve reliability of 5-HT measurement. Here, a model was developed to track 5-HT electrode adsorption and resulting current output by combining Langmuir adsorption kinetic equations and adsorption-limited electrochemical equations. 5-HT adsorption binding parameters were experimentally determined at a carbon-nanotube coated Au electrode: K_D_ = 7 × 10^−7^ M, k_on_ = 130 M^−1^ s^−1^, k_off_ = 9.1 × 10^−5^ s^−1^. A computational model of 5-HT adsorption was then constructed, which could effectively predict 5-HT fouling over 50 measurements (R^2^ = 0.9947), as well as predict electrode responses over varying concentrations and measurement times. The model aided in optimizing the measurement of 5-HT secreted from a model enterochromaffin cell line—RIN14B—minimizing measurement time. The presented model simplified and improved the characterization of 5-HT detection at the selected electrode. This could be applied to many other adsorption-limited electrochemical analytes and electrode types, contributing to the improvement of application-specific modeling and optimization processes.

## 1. Introduction

Serotonin (5-hydroxytryptamine, 5-HT) is an essential neurotransmitter underlying both neurological and gastrointestinal (GI) functions, making it a target of study for these functions and their related disorders. In the brain, 5-HT helps regulate behaviors such as sleep and learning [1,2] as well as onset of anxiety and depression [3]. 5-HT is produced at even higher levels in the mammalian GI tract in pathways responsible for peristalsis [4], visceral sensation [5], and symptoms such as nausea [6], and has been implicated in a number of functional GI disorders [7,8,9,10]. Often, the goal is to quantify 5-HT concentrations within tissues or bodily fluids and correlate this with biological processes, especially underlying disease. To this end, there is a long history of electrochemical 5-HT detection methods, which provide real-time, label-free quantification of 5-HT within these biological environments, given adequate accessibility for the insertion of electrodes [11,12,13] in benchtop and in vivo environments.

5-HT electrochemical detection is an adsorption-limited reaction [14], where 5-HT molecules adsorb to an electroactive surface and are oxidized, producing a measurable current signal. This implies that 5-HT binding and detection are time-dependent, requiring strict control over the measurement frequency to record an accurate concentration [15,16]. Furthermore, electrochemical detection leads to downstream fouling as spontaneous 5-HT oxidative polymerization results in the formation of polymeric byproducts [17], which cover the electrode and decrease electroactive surface area over successive measurements [14,18,19]. 5-HT fouling causes progressive loss of sensitivity over time, which limits the reliability of 5-HT quantification. There is significant research towards optimizing electrode materials to resist fouling (e.g., carbon nanotubes (CNTs) [20,21], diamond [22,23]) or electrochemical protocols that discourage fouling (e.g., fast-scan cyclic voltammetry [13,24,25]). However, these methods rarely fully eliminate fouling, and thus there remains a level of unreliability in the repeatability of 5-HT sensing. The Langmuir isotherm model of adsorption [26] is used to study adsorption of molecules, such as 5-HT, onto substrates, such as electrodes. This type of model is commonly used to quantify electrode sensitivity (slope of linear region) and linear range, but is less frequently used to determine fundamental binding parameters underlying electrochemical detection [27]. Furthermore, the Langmuir model of adsorption has never been directly combined with adsorption-limited electrochemical equations which describe the direct transfer of electrons from redox molecule to electrode, measuring current in direct correlation with molecular concentration [28].

Here, we aim to develop a simple but effective method to model 5-HT electrochemical detection, based on experimentally-determined binding parameters with a given electrode, derived from a combination of Langmuir and electrochemical adsorption equations. 5-HT measurements made with a CNT-modified electrode, used here to demonstrate the method, were fitted with both equilibrium and time-dependent Langmuir adsorption models to characterize 5-HT binding affinity with the electrode. Langmuir equations combined with adsorption-limited electrochemical equations were then used to model 5-HT binding and subsequent current signal generation, which could effectively predict electrochemical signal output based on 5-HT concentration and accumulation time between measurements. These equations and binding parameters were also used to track the process of 5-HT fouling at the electrode, constituting a computational model of 5-HT adsorption, detection, and fouling. This model also aids in optimizing sensing parameters for a given application, such as detection of 5-HT from RIN14B cells, an immortalized cell line which expresses a similar 5-HT secretion pathway to enterochromaffin cells in the GI tract [29]. The model demonstrated here could be applied to any electrode system given characterization of its unique binding parameters with 5-HT, or other adsorption-limited redox molecules, and could be combined with models of complex biological environments to improve understanding of molecular behavior in tissues of interest.

## 2. Materials and Methods

### 2.1. Adsorption Model Development

**Figure 1:**
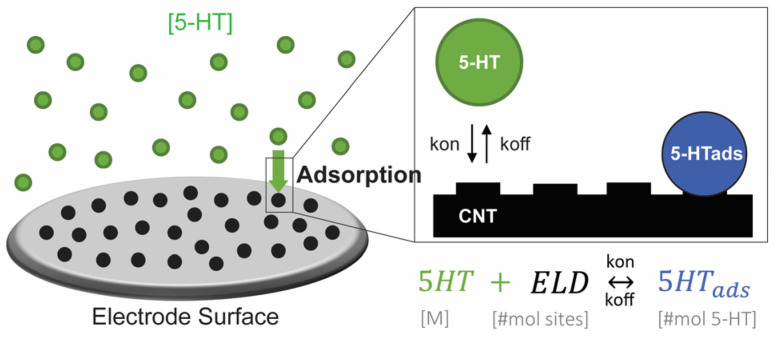
Schematic of 5-HT molecules binding to a surface with available electrode (ELD) binding sites, shown as raised sections of the black CNT surface. This can be explained by the labeled adsorption reaction.

Langmuir Adsorption Kinetics are generally used to define the interaction of a ligand and a substrate in order to understand the formation of a product under equilibrium and time-dependent conditions. Here, they can describe the adsorption-limited electrochemical detection of 5-HT, which here will be demonstrated using cyclic voltammetry (CV). Detection begins with 5-HT adsorption onto the electrode surface to form the product 5-HT_ads_, the number of moles of 5-HT adsorbed on the electrode, as illustrated in Figure 1. This binding reaction states:

(1) $$5-\mathrm{HT}\;+\;\mathrm{ELD}\;↔\;5-{\mathrm{HT}}_{\mathrm{ads}}$$

where 5-HT is the concentration of free 5-HT in aqueous solution (M), ELD is the number of available binding sites on the electrode surface (#mol sites), and 5-HT_ads_ is the number of adsorbed 5-HT (#mol). Modeling the electrode surface with a discrete number of binding sites, rather than surface area, allows for modeling of one-to-one binding with molecules, as is described by Langmuir kinetic processes.

The forward and reverse reaction rates are k_on_ (M^−1^ s^−1^) and k_off_ (s^−1^), respectively. The dissociation constant, K_D_ (M), can be found from the ratio of k_off_/k_on_, and will be used here to define binding affinity. Of note, we can assume the binding reaction has reached equilibrium when sufficient accumulation time (t_acc_) is given to achieve 5-HT binding saturation on the electrode. One can follow the derivation included in the Supplementary Information (Supplementary Figure S1) to determine the following Langmuir isotherm equation at equilibrium [26]:

(2) $$5-{\mathrm{HT}}_{\mathrm{ads},\mathrm{eq}}=\frac{\left[5-\mathrm{HT}\right]\cdot {\mathrm{ELD}}_{\mathrm{tot}}}{K_{D}+\left[5-\mathrm{HT}\right]}$$

where ELD_tot_ is the total number of available binding sites on the electrode surface. This equation can be fitted to experimental data in order to calculate key values for any given electrode and analyte: K_D_ and ELD_tot_. 

The following equations describe time-dependent adsorption and desorption, respectively, based on the same Langmuir isotherm model [30]:

(3) $$5-{\mathrm{HT}}_{\mathrm{ads}}\left(t_{\mathrm{acc}}\right)=5-{\mathrm{HT}}_{\mathrm{ads},\mathrm{eq}}\left(1-e^{-\left(\left[5-\mathrm{HT}\right]\cdot k_{\mathrm{on}}+k_{\mathrm{off}}\right)\cdot t_{\mathrm{acc}}}\right)$$

(4) $$5-{\mathrm{HT}}_{\mathrm{ads}}\left(t_{\mathrm{acc}}\right)=5-{\mathrm{HT}}_{\mathrm{ads},\mathrm{eq}}\;\cdot e^{\left(-t_{\mathrm{acc}}\cdot k_{\mathrm{off}}\right)}$$

where 5-HT_ads_ is calculated for a given applied t_acc_ (s) of binding time. The adsorption regime is considered when a constant non-zero [5-HT] is applied to the electrode, resulting in progression of the binding reaction toward equilibrium. The desorption regime is when [5-HT] = 0, resulting in unbinding toward full dissociation of 5-HT from the surface. Here, Equation (3) is fitted with experimental cyclic voltammetry (CV) data measuring a constant [5-HT] over increasing t_acc_ to determine k_on_ and k_off_, given the solution of 5-HT_ads,eq_ from (2) under equilibrium conditions. Equations (3) and (4) are later modeled using these calculated binding parameters to predict 5-HT binding behavior at various binding times and concentrations.

Adsorption-controlled electrochemical processes are governed by the binding of a given redox-active analyte (such as 5-HT) to the electrode surface, wherein the surface coverage of these redox molecules leads to direct electron transfer that causes an increase in anodic peak current (Ipa) signal. This interaction can be described by the following equation [28]:

(5) $$I_{\mathrm{pa}}=\frac{n^{2}F^{2}}{4\mathrm{RT}}\cdot \upsilon \cdot A\cdot \Gamma$$

where n is the number of electrons transferred per molecule, F is Faraday’s constant, R is the ideal gas constant, T is temperature (K), ν is scan rate, A is surface area, and Γ is molecular surface coverage. Here, A can be described as the surface area of the electrode (mm^2^) and Γ is the molecular surface coverage of 5-HT $\left(\frac{5-HT_{\mathrm{ads}}}{A}\right)$. The product of A∙Γ reduces to 5-HT_ads_, which linearly scales with Ipa. Therefore, Equation (5) reduces to Equation (6):

(6) $$I_{\mathrm{pa}}\left(t_{\mathrm{acc}}\right)=\frac{n^{2}F^{2}}{4\mathrm{RT}}\cdot \upsilon \cdot 5-{\mathrm{HT}}_{\mathrm{ads}}\left(t_{\mathrm{acc}}\right)$$

This equation serves as a conversion between the number of adsorbed moles of 5-HT (5-HT_ads_), which can be calculated using Equations (2)–(4), and electrochemical signal output (Ipa). It should be noted that this equation is time-dependent in that 5-HT_ads_ depends on t_acc_, and thus so does Ipa. If the applied t_acc_ is enough to achieve equilibrium binding, then 5-HT_ads_(t_acc_) = 5-HT_ads,eq_.

If one were modeling electrochemical detection of an adsorbed analyte, they could follow the above equations in the presented order to estimate the signal that may be obtained from detection of a given analyte, with known concentration over a given t_acc_. Conversely, the equations may be used in reverse order to calculate the analyte concentration measured from a given electrochemical experiment, given its Ipa signal and t_acc_. Furthermore, the intermediate calculations of 5-HT_ads_ allows for more in depth modeling of molecular behavior, as might be useful in complex biological environments.

5-HT Fouling can also be modeled using these equations. The fouling process can be identified as when CV signal decreases, even in a constant 5-HT concentration solution, due to incremental covering of the electrode surface with polymerized 5-HT material. This can be approximated as a decrease in available ELD binding sites after each 5-HT CV measurement, wherein some fraction of bound 5-HT molecules do not unbind. The following equation is proposed to describe this fouling of the ELD surface:

(7) $${\mathrm{ELD}}_{i+1}={\mathrm{ELD}}_{i}-a\cdot \left(5-{\mathrm{HT}}_{\mathrm{ads},i}\right)$$

where i denotes CV cycle number, ELD_1_ = ELD_tot_, and *a* is a proportionality factor describing the fraction of 5-HT_ads_ that does not unbind and therefore fouls ELD sites. In this way, the rate of fouling is proportional to 5-HT_ads_, which depends on 5-HT concentration and applied t_acc_, as has been found experimentally (Supplementary Figure S2).

A model was established to fit this fouling process to experimental CV data. For each CV cycle, Equation (2) was used to calculate the equilibrium 5-HT_ads,eq_ for the given [5-HT] and available ELD sites (ELD_i_), which was used in Equation (3) to calculate the time-dependent 5-HT_ads_ given the applied t_acc_. Then, Equation (7) was used to calculate the decrease in total available ELD sites, where *a* was solved to fit the experimental data, and the calculated ELD_i+1_ value was used for the next CV cycle. Finally, 5-HT_ads_ was converted to Ipa for each cycle using Equation (6), and both were plotted over CV cycle.

### 2.2. Electrode Fabrication

**Figure 2:**
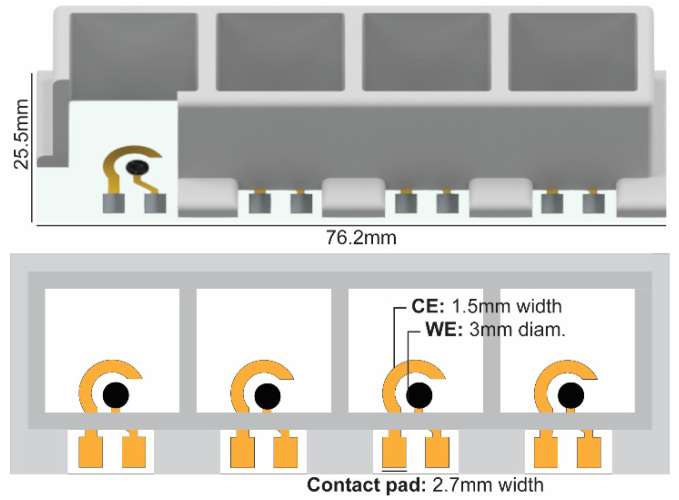
Schematic of Au-CNT electrode platform, including (**top**) CAD diagram of assembly and (**bottom**) diagram of electrode layout.

Here, an electrochemical platform was fabricated consisting of Au electrodes modified with CNTs (Au-CNT) to increase electrode sensitivity to 5-HT, and potentially limit fouling [16,19]. This Au-CNT electrode was previously demonstrated to exhibit adsorption-limited detection of 5-HT, given by a linear relation between measured Ipa and applied scan rate [15]. The electrochemical testing platform was assembled by fabricating Au-CNT electrodes on a standard 25.4 × 76.2 mm glass slide and then affixed with a 3D-printed well housing, materials which are relatively inert and robust (Figure 2). The glass slides were solvent cleaned with acetone, methanol, and isopropanol (AMI), followed by a DI water rinse. Au working and counter electrodes (WE, CE) were patterned on the glass via electron beam evaporation (20 nm Ti/100 nm Au) through a laser-cut shadow mask, as previously described [15,31]. The WE was modified with 1 mg/mL purified single walled CNTs (Carbon Solutions Inc., Riverside, CA, USA) dispersed in a 1:1 mixture of ethanol and N-methyl-2-pyrrolidone (NMP) (Thermo Fisher Scientific, Waltham, MA, USA). The solution was ultrasonicated to improve uniformity, and then 3 µL was drop cast on the WE after being heated on a 200 °C hotplate, which functions to evaporate the edge of the CNT droplet before it spreads across the surface of the glass, containing the coating to the WE. This method was previously shown to produce a thin, uniform mesh of CNTs on the electrode surface, which did not visually degrade over the process of experimentation [15]. The whole slide was then cleaned again with AMI before assembly with the 3D printed wells.

The wells were designed in Autodesk^®^ Inventor™ and printed with an Objet500 Connex3 3D printer (Stratasys, Eden Prairie, MN, USA) with MED610, a photopolymer using PolyJet technology. MED610 is a bioinert 3D printing material, characterized to not show significant cytotoxicity by ISO standards if proper cleaning procedures are taken [32]. This cleaning included support material removal by sonication in 2% NaOH and 1% Na_2_SiO_3_ for up to 24 h, followed by thorough leeching of any further photoreactive materials by alternating sonication in isopropanol and diH_2_O for 2 h each [33]. Once dried, the rim of the 3D printed well structure was coated with polydimethylsulfoxide (PDMS) in a 10:1 ratio (Sylgard^®^184, Dow Corning, Midland, MI, USA), affixed to the glass slide, and thermally cured on an 80 °C hotplate for 1 h. This leaves a 10 mm space in the rim of the 3D printed wells to provide access to the contact pads for wiring via microalligator clips.

### 2.3. Electrochemical Methods

5-HT was purchased from Alfa Aesar (Ward Hill, MA, USA) and dissolved in 1× phosphate buffered saline (PBS, pH = 7.4) (Thermo Fisher Scientific) for electrode characterization. All electrochemical experiments were carried out using the VSP-300 potentiostat, and data was recorded using the EC-Lab software, both from Bio-Logic Science Instruments (Seyssinet-Pariset, France). Cyclic voltammetry (CV) measurements of 5-HT were performed between 0.1–0.5 V to center around the 5-HT redox potential at ~0.33 V. These measurements were performed using the glass slide-fabricated Au CE and Au-CNT WE, and a standard Ag/AgCl reference electrode (RE) in saturated KCl (CH Instruments, Austin, TX, USA) that was manually inserted into the fluid during electrochemical measurements. This electrode was chosen for its superior longevity compared to thin-film Ag electrodes, which can drift over time [34].

Because of the adsorption-limited nature of 5-HT electrochemical detection, an accumulation time (t_acc_) was applied before each CV cycle, holding the electrode at open circuit potential for a given time to allow for sufficient 5-HT adsorption to produce a measurable peak, or to reach equilibrium binding. Supplementary Figure S3 shows a comparison of different scan rates and t_acc_s used to detect 100 nM 5-HT. While a more in-depth analysis of t_acc_-dependent detection is performed later, this analysis showed that compared to lower scan rates, 400 mV/s resulted in the least amount of noise in the current while still maintaining peak sharpness at low concentrations. Thus, CV measurements were performed at 400 mV/s, unless otherwise stated.

MATLAB (MathWorks, Natick, MA, USA) was used to process the CV data, in which current was plotted against potential (I vs. E). The data was first smoothed using a low-pass filter with a cutoff frequency of 3 Hz and a sampling frequency of 2 kHz. To extract anodic peak currents (Ipa), a linear regression was fitted against the background signal preceding each CV peak and then subtracted from the measured current peak (Supplementary Figure S4).

### 2.4. Cell Culture Testing

The RIN14B rat islet cell line (ATCC^®^ CRL2059TM, Manassas, VA, USA) was used as a surrogate for 5-HT-secreting enterochromaffin cells in the mammalian gut epithelium. Cells were maintained in T25 polystyrene flasks with incubation at 37 °C and 5% CO_2_ in Dulbecco’s Modified Eagle Medium (DMEM) with 10% fetal bovine serum (FBS) (Thermo Fisher Scientific). Cell passaging was performed every 3–5 days with 2.5% trypsin-EDTA (Sigma-Aldrich, St. Louis, MO, USA).

Cells were grown until confluency (~63 × 10^5^ cell count) and then stimulated to release 5-HT using allyl isothiocyanate (AITC) and ionomycin (Thermo Fisher Scientific). AITC was purchased as an oil-based solution, which was first diluted in DMSO, then diluted further in diH_2_O to result in a 3 mM AITC stock solution in 30% DMSO. To perform cell stimulation, cells were first washed with PBS pre-warmed to 37 °C to avoid premature stimulation. Then, 5-HT release was stimulated by incubating cells for 20 min in 1 mL stimulation solution containing either 300 µM AITC or 10 µM ionomycin. This stimulation solution consisted of 4 °C high-glucose DMEM, containing 1.8 mM CaCl_2_, 25 mM glucose, and 2 µM fluoxetine, which are commonly used in RIN14B literature to boost the cells’ response to molecular triggers and maximize 5-HT secretion over the incubation period [29,35,36]. Supernatant was then transferred to an Au-CNT electrode to detect 5-HT, using a 30 min t_acc_ and 50 mV/s scan rate. Electrodes were shown to have no response to AITC or ionomycin alone (Supplementary Figure S5).

Supplementary Figure S6 shows the electrode sensitivity to 5-HT in comparison to other common redox molecules including uric acid, ascorbic acid, and dopamine (Sigma-Aldrich). In the performed potential range, the electrodes showed no response to uric acid or ascorbic acid. Dopamine produced an oxidation peak at 0.23 V, well-separated from 5-HT at 0.37 V, but with a lower Ipa (3.3 µA and 4.3 µA, respectively). When all molecules were combined, the 5-HT peak was still clearly distinguishable.

## 3. Results

### 3.1. Adsorption Parameter Determination

#### 3.1.1. Equilibrium Adsorption Parameters

**Figure 3:**
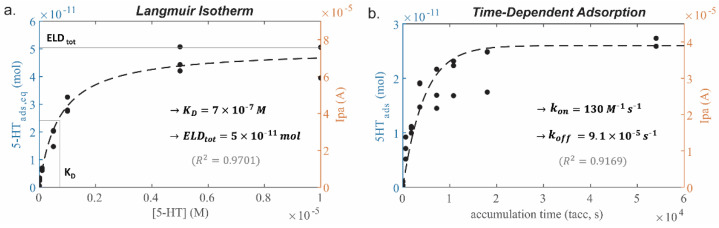
Experimental characterization of 5-HT adsorption. (**a**) Langmuir isotherm constructed by measuring [5-HT] = 0.01–10 µM, accumulation time (t_acc_) = 15 h, where parameters ELD_tot_ and K_D_ can be fit to Equation (2) and plotted as the dashed black curve. (**b**) Graph of time-dependent 5-HT adsorption over t_acc_ = 1 min–15 h, [5-HT] = 1 µM, where parameters k_on_ and k_off_ can be fitted to Equation (3) and plotted as the dashed black curve. (**a**,**b**) Both graphs are plotted with equivalent values of 5-HT_ads_ and Ipa on the left and right *y* axes, respectively, and the models were fitted using the 5-HT_ads_ values. Labeled R^2^ denotes standard deviation.

To determine 5-HT adsorption parameters for a given electrode, under equilibrium state, such as K_D_ and ELD_tot_, experimental Ipa measurements were plotted over increasing [5-HT] to construct a Langmuir isotherm (Figure 3a). A t_acc_ of 15 h was used to ensure equilibrium binding, as confirmed by the saturation time in Figure 3b, and each data point was measured with a fresh electrode to avoid the influence of fouling. The measured Ipa data was converted to 5-HT_ads_ using equation (6), and both were plotted as y axes. Equation (2) was fitted to this data to calculate ELD_tot_ = 5 × 10^−11^ mol and K_D_ = 7 × 10^−7^ M (R^2^ = 0.9701). The latter indicates a sub-micromolar affinity of 5-HT with this Au-CNT electrode. This is on par with binding probes of modest affinity [37], but many orders of magnitude lower affinity than the strongest binding molecules (e.g., biotin-streptavidin, K_D_~10^−14^ M [38]).

#### 3.1.2. Time-Dependent Adsorption Parameters

Time-dependent 5-HT adsorption parameters, such as k_on_ and k_off_, can be experimentally determined by measuring a constant [5-HT] solution (1 µM) with increasing applied t_acc_ (Figure 3b). Again, fresh electrodes were used for each measurement, and extracted Ipa and 5-HT_ads_ values were plotted for comparison. Equation (3) was fitted, using the average 5-HT_ads,eq_ value from the 1 µM data points in Figure 3a (5-HT_ads,eq_ = 2.6 × 10^−11^ mol), to obtain k_on_ = 130 M^−1^ s^−1^ and k_off_ = 9.1 × 10^−5^ s^−1^ (R^2^ = 0.9169). Determination of these time-dependent binding parameters allows for computational estimation of the signal generated from a 5-HT measurement at this Au-CNT electrode given any 5-HT concentration or applied t_acc_.

Increased variation in the measured signal likely resulted from slight variations introduced when drop-casting CNT onto electrodes, reducing the possible R^2^ values. However, this variation is minimal, and both graphs were able to be fitted with Langmuir isotherm models of adsorption, resulting in estimation of the average binding site availability and adsorption kinetic parameters for all electrode surfaces.

### 3.2. Adsorption Model Results

**Figure 4:**
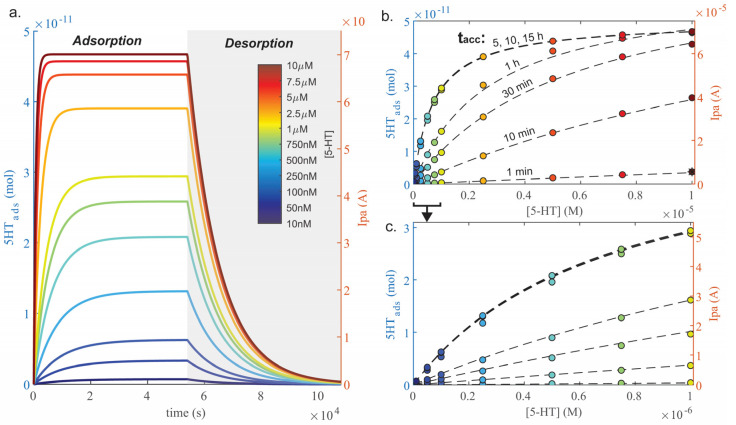
(**a**) Computationally-constructed model of 5-HT adsorption and desorption on the Au-CNT electrode based on Equations (3) and (4) over a range of physiologically relevant concentrations. (**b**) Sensitivity curves plotted from datapoints in (**a**) where color corresponds to [5-HT]. Curves are plotted comparing different t_acc_s, as labeled. (**c**) Sensitivity curves from (**b**) zoomed around 10 nM–1 µM 5-HT.

Given the estimation of the average number of electrode binding sites (ELD_tot_), as well as 5-HT–ELD binding parameters (K_D_, k_on_, and k_off_), a model was constructed using Equations (2)–(4) to computationally predict 5-HT adsorption and desorption over time at different concentration levels (Figure 4a). The model fits the expected shape of adsorption and desorption graphs [29], reaching a plateau at an equilibrium binding level (5-HT_ads,eq_) during the adsorption phase, and decreasing to zero during the desorption phase. The adsorption phase of the graph provides insights about the signal achieved over a given adsorption time (t_acc_), which plateaus to a maximum value depending on concentration. For instance, over 15 h, the lowest concentrations (10–50 nM 5-HT) barely reach a binding equilibrium, while the highest concentrations equilibrate within 1–2 h (5–10 µM 5-HT). 

These computational estimates can be used to predict experimental results. For instance, plotting calculated Ipa or 5-HT_ads_ over [5-HT] can estimate expected electrode sensitivity and linear range at various t_acc_s (Figure 4b,c), and these values are listed in Table 1. Interestingly, when considering sensitivity, the electrode reaches its effective maximum sensitivity at t_acc_ = 5 h, even though the equilibrium saturation time is 15 h. The subsequent increase in sensitivity is not proportional to the increase in t_acc_, with diminishing returns over t_acc_ = 1 h. The same can be said for the linear range, which was visually determined. Increasing t_acc_ past 1 h does not drastically increase the ability to detect [5-HT] lower than 50 nM. In fact, when the graph is zoomed around lower concentrations (Figure 4c), the signal begins to level off at 500 nM at t_acc_ ≥ 5 h, indicating a smaller linear range and a decreased capability to detect sub-micromolar concentrations compared to shorter t_acc_s. This presents the ability to estimate the optimal experimental t_acc_ needed to detect a given range of 5-HT. For instance, RIN14B cell 5-HT secretion is expected to be ~100 nM, thus only t_acc_ = 30 min should be needed to detect this concentration. Therefore, the remaining experiments will characterize electrode functions using t_acc_ = 30 min to minimize experimental time.

### 3.3. Tracking 5-HT Fouling

**Figure 5:**
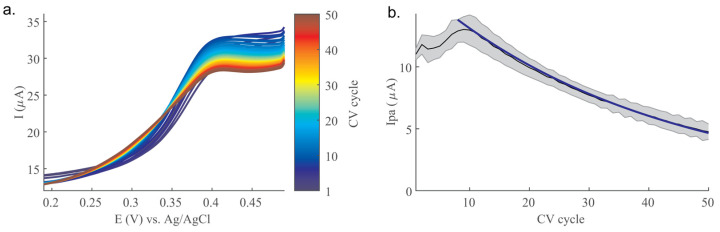
Evaluation of Au-CNT electrode fouling over 50 CV cycles in 500 nM 5-HT (t_acc_ = 30 min). (**a**) CV cycles showing incrementally decreasing peak height. (**b**) Plot of CV Ipa from 4 Au-CNT electrodes, where the black line indicates average Ipa and the shaded area indicates standard deviation (*n* = 4). Blue line indicates adsorption model-based fit to fouling process (R^2^ = 0.9947 over cycles 7–50). Model excludes initial rise phase in Ipa signal.

Electrode fouling was experimentally induced by measuring 500 nM 5-HT over 50 CV cycles. Figure 5a shows the CV cycles over this process for a representative Au-CNT electrode, where current peaks incrementally decrease. Figure 5b plots the average Ipa measured across the electrodes (*n* = 4). Interestingly, Ipa initially increases (cycle 1–7), after which Ipa steadily decreases (cycle 7 onward). Figure S7 shows that electrode reproducibility also decreases over the course of fouling, measured as reproducibility standard deviation (RSD), which increases from <10% to >15% from cycle 1 to 50.

The fouling model was fitted to this decreasing phase, shown as the blue line in Figure 5b, using Equation (7). The best fit occurred with a = 0.25, meaning that 25% of bound 5-HT molecules would go on to foul the electrode after each CV cycle. The fit corresponding to the decreasing portion of the experimental data (cycle 7–50) was very good (R^2^ = 0.9947) when a proportionality factor of 1.8× was applied to Equation (6), the conversion of 5-HT_ads_ to Ipa, which is not accounted for by any existing parameters in the equation. This suggests that the observed signal is slightly higher than may be predicted by the solved adsorption parameters. The implications of this phenomenon will be expanded on in the discussion.

### 3.4. Prediction of 5-HT Detection

**Figure 6:**
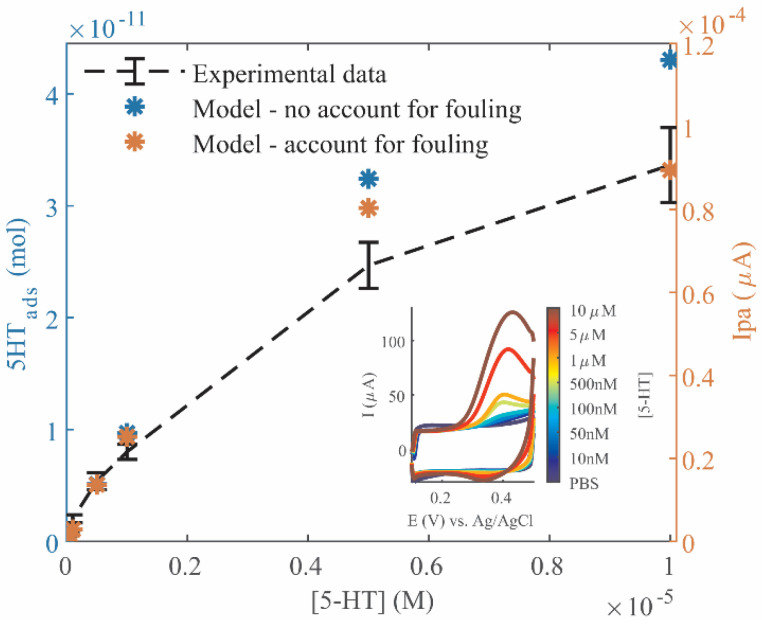
Sensitivity curve of 5-HT measured over concentrations 0.01–10 µM (t_acc_ = 30 min), indicated by the dashed black line (*n* = 4 electrodes). Error bars indicate standard deviation. Colored markers indicate model-predicted Ipa values, accounting for fouling (orange) or not (blue). Accounting for fouling improves model fit with data (R^2^ = 0.9713 vs. R^2^ = 0.8524). Inset: Representative CV curves of measured data.

This model was then applied to predict increasing 5-HT concentrations. A sensitivity curve was experimentally constructed using Au-CNT electrodes. Figure 6 shows the average experimental Ipa measurements for [5-HT] = 0.01–10 µM (black dashed line), where the model was applied using Equations (2) and (3) to predict experimental results, with and without accounting for fouling (orange and blue, respectively). Again, a 1.8× factor applied to Equation (6) resulted in a better fit of calculated and experimental Ipa values. In this case, as opposed to Figure 3, each electrode was used to measure the full range of 5-HT concentrations, so there would be some expected fouling in this process. The model that does not account for fouling calculated the last two data points at much higher values than the experimental averages, and the overall fit suffers (R^2^ = 0.8524). However, when fouling is accounted for in the model, the difference in these last two data points is much less, and the fit becomes much better (R^2^ = 0.9713). This is an example of how this adsorption model can be used to predict electrode detection of changing 5-HT concentrations. The predicted values do not fully match the experimental values, so more analysis may be necessary to develop this model to account for the complex chemical reactions occurring during fouling. However, the general trend is nicely predicted by the model, and serves as a starting point to predict 5-HT adsorption and fouling.

The linear portion of the experimental sensitivity curve can be analyzed to estimate electrochemical response parameters. The linear range is within 0.1–1 µM 5-HT, where slope denotes a sensitivity of 17.8 A/M (R^2^ = 0.9815). Electrode resolution is calculated as 3σ = 501 nA, where σ is the standard deviation of the lowest concentration (10 nM). Limit of detection (LOD) and limit of quantification (LOQ) are calculated as 3σ/sensitivity and 10σ/sensitivity, respectively, equaling 28.1 nM and 93.8 nM, confirming that 5-HT is quantifiable down to ~100 nM at t_acc_ = 30 min. The sensitivity predicted in Figure 4b for t_acc_ = 30 min is 11.8 A/M, and applying the 1.8× proportionality factor, is 21.2 A/M. The experimental and predicted sensitivities are within ±3.4 A/M (~19%), and the linear range fits exactly with the model, giving further confidence to its validity.

### 3.5. Detection of Cell Released 5-HT

**Figure 7:**
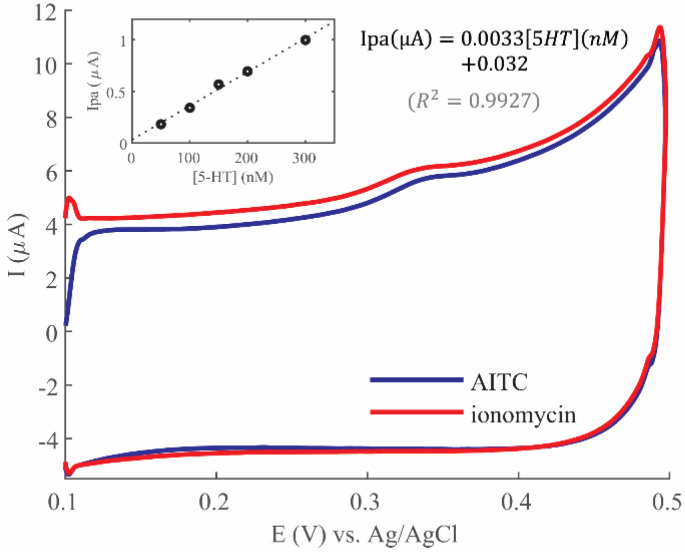
CV detection of 5-HT in RIN14B cell supernatant, t_acc_ = 30 min, scan rate = 50 mV/s. Inset: Electrode calibration used to calculate measured 5-HT from cells via displayed equation.

Finally, the model developed here has the potential to help optimize electrochemical detection parameters for various applications. One such application is the detection of 5-HT released from in vitro cell cultures under applied chemical stimulation, such as AITC, a TRPA1 receptor agonist involved in visceral pain [5,29], and ionomycin, a Ca^2+^ ionophore which diffuses into cells to trigger a Ca^2+^ signaling cascade [29,39], both of which result in vesicle-mediated 5-HT release. RIN14B cells are known to release 5-HT in the ~100 nM range in response to these chemicals [29,36]. Therefore, based on the modeling results in Figure 4, t_acc_ = 30 min was selected for electrochemical detection of 5-HT release from RIN14B to minimize measurement time while achieving the desired LOD. As shown in Figure 7, CV peaks were detected in the cell supernatant after stimulation, centered at Epa~0.31–0.33 V, corresponding to the expected redox potential of 5-HT. These peaks had Ipa values of 0.89 µA and 1.05 µA for ionomycin and AITC, respectively. After calibration of the electrode (Figure 7, inset), these Ipa values were converted to [5-HT]: ionomycin: 260 nM, AITC: 308 nM 5-HT. These 5-HT concentrations are within the expected range secreted from RIN14B cells, in a fraction of the time needed to perform ELISA, as is the standard method. The detected 5-HT concentrations are significantly higher than the LOQ, making the measurement reliably quantifiable. This demonstrates the benefit of using modeling to estimate the minimum t_acc_ required of this electrode, given expected concentrations. A model such as this can further aid in developing a protocol in the future to track 5-HT secretion over time from these cells, which would be more limited using laborious methods such as ELISA due to long incubation times.

## 4. Discussion

In this work, we demonstrated the utility of an expanded adsorption model to predict and characterize electrode detection of 5-HT. To our knowledge, this is the first model which combines Langmuir and electrochemical adsorption models, and the first to calculate the binding affinity of a redox molecule to a fabricated electrode. Whereas most electrochemical electrodes are quantitatively compared by their sensitivity, LOD, and linear range, these parameters can depend on other factors such as t_acc_ and fouling behavior and thus do not fully characterize their function. K_D_ is a more fundamental parameter of the binding reaction, and thus may present a more useful quantitative factor for comparison between electrodes, such as when modifying a given electrode surface to deliberately increase molecular binding.

Using fabricated Au-CNT electrodes for this proof of concept, we experimentally determined adsorption parameters of 5-HT binding to this electrode. These parameters were then used to construct a computational model of 5-HT adsorption and desorption using adsorption-limited electrochemical and Langmuir adsorption equations. This model is useful for optimizing measurement conditions for a given application, such as determining electrode sensitivity across different t_acc_ values. A model of electrochemical 5-HT fouling was then proposed, in which 5-HT adsorption and oxidation by CV results in some fraction of electrode binding sites being irrevocably covered by oxidized 5-HT byproducts. Although this is a simple model that does not account for specific chemical processes of fouling, the model was able to approximate the rate of fouling very closely when averaged across four electrodes. Interestingly, the electrodes exhibited an initial phase of signal increase over the first several CV cycles, which is not accounted for in the model and therefore was not fitted here. This could consist of a buildup of bound 5-HT that does not completely oxidize in the first several cycles, or a temporary increase in binding affinity of free 5-HT with a low density of oxidized 5-HT byproducts. This model could be expanded in the future to include this initial phase, given more precise understanding of the chemical interactions in this process. 

Furthermore, it was noted that because of this effect, fresh electrodes exhibited slightly lower affinity for 5-HT than electrodes which had been previously exposed to low concentrations of 5-HT. This may also explain why, when converting model-calculated 5-HT_ads_ to Ipa using Equation (6), a factor of 1.8× resulted in a precise fit of the modeled and experimental data for both Figure 5 and Figure 6. This would suggest that a higher Ipa signal is obtained than predicted for a given number of adsorbed 5-HT molecules. Here, this proportionality factor was able to account for the difference, but this has yet to be experimentally confirmed. In addition, this effect can be leveraged in the future to increase electrode sensitivity by pre-conditioning electrodes with 5-HT, wherein the experimental adsorption parameters can be compared to fresh electrodes.

This discrepancy between model-calculated 5-HT_ads_ and measured Ipa signal could also indicate that that an adsorption model cannot capture all possible mechanisms of 5-HT electrochemical detection. Electron transfer from a redox analyte to an electrochemical electrode can occur though quantum-mechanical electron tunneling across a nanometer-scale distance, bypassing the need for physical molecular adsorption. This can even occur across an insulating layer, such as fouled material [40]. Our electrodes are modified with CNTs functionalized with carboxylic acid groups, both of which are capable of increasing electron transfer rates and decreasing the electron tunneling distance [41,42], suggesting this as a possible mechanism of detection for this electrode. Therefore, future work will be dedicated to evaluating the effect of any possible non-adsorptive 5-HT detection mechanisms, in addition to the adsorption mechanism addressed here. It should be noted, however, that 5-HT detection is reported to be adsorption-limited [14], suggesting that this mechanism is predominant and can mostly predict electrochemical signal generation.

The model was also used to predict electrochemical signal over a range of 5-HT concentrations. When the model accounted for fouling, the fit of the model to the data increased. These results indicate that the model of 5-HT adsorption could accurately predict both electrochemical detection and fouling, to a useful extent, and that the calculated adsorption kinetic parameters worked for multiple experiments using different electrodes.

Measurement of 5-HT release from RIN14B cells, which has been commonly performed using ELISA as a gold standard in literature [29,35,36], was successfully reproduced here using our electrochemical method. The model and the experimental sensitivity curve aided in quantifying 5-HT release from RIN14B cells, using the selected minimum t_acc_ of 30 min. The measurement of ~200–300 nM 5-HT lies above the electrode’s LOQ. This measurement corresponds to ~4–8 pmol 5-HT released per 10^5^ cells, which is very similar to estimations from literature (~7 pmol/10^5^ cells by Nozawa, et al. [29]) This demonstrates the feasibility of using electrochemical methods for 5-HT detection from these cells, which will, in the future, be refined and validated in a comparative study side-by-side with ELISA upon the development of a desired measurement protocol. We aim to achieve temporal tracking of 5-HT release under applied conditions by leveraging shorter measurement times compared to ELISA, as this is an integral benefit of electrochemical methods. This would also require the anticipation of 5-HT fouling, which is demonstrated in this paper. Further cell measurements would require that additional redox-active cellular byproducts be tested for their potential interference with these 5-HT measurements, such as H_2_O_2_ and other reactive oxygen species that are markers of oxidative stress [43,44].

Future work should aim to improve upon these results to achieve a more robust understanding of adsorption-limited electrochemical detection mechanisms and improve sensor performance. The use of pulsed voltametric techniques (e.g., ACV, DPV) can increase resolution and sensitivity of detection, which may be necessary to further elucidate dynamics of molecular binding. Potential sources of error should be investigated as well to improve our model’s reliability. For instance, the stability of the CNT dropcast layer should be evaluated over the course of repeated electrochemical measurements, as CNTs can be shed in this environment. High-precision spectroscopic methods may be necessary to identify microscale-level changes to the CNT surface, which may be a source of variability.

The computational method developed here can be used to determine adsorption parameters of analyte binding to an electrode and conversion to electrochemical signal, aiding in precise modeling of molecular behavior. It can be used to evaluate adsorption-based electrochemical detection at any given electrode where binding with a redox molecule occurs, such as to quantitatively compare binding affinity between electrodes with experimentally modified surfaces. For instance, this could be used to quantify the difference in binding affinity between a bare Au electrode and one modified with carbon materials, as it has been shown that the presence of a modified carbon surface dramatically increases 5-HT binding on Au electrodes [15]. This can also be applied to models of complex biological environments, taking into account environmental factors such as fluid flow, volumes, tissue porosity, etc. It is necessary, in these complex environments, to understand the context of measured molecular concentrations, since concentration depends on volume, which can vary drastically. Knowing the fundamental binding kinetics of a redox molecule with any given electrode, under an adsorption-limited scheme, allows back-calculation of unknown concentrations based on the physics of the interaction, given an understanding of the physics of the environment. Therefore, the methods presented here can act as a useful resource for many studies where molecular adsorption is the foundation of quantitative measurement.

## Figures and Tables

**Table 1:** Sensitivity and linear range calculated from adsorption model, based on Figure 4b.

t_acc_	Sensitivity	Linear Range
1 min	0.55 A/M	1–10 µM
10 min	4.67 A/M	0.25–5 µM
30 min	11.8 A/M	0.1–2.5 µM
1 h	23.7 A/M	0.05–1 µM
5, 10, 15 h	41 A/M	0.05–0.5 µM

## Data Availability

Data available upon request.

## Supplementary Materials

The following supporting information can be downloaded at: https://www.mdpi.com/article/10.3390/mps6010006/s1, Figure S1, Sample Langmuir isotherm. Black dots show experimental data plotted as 5-HT_ads,eq_ against [5-HT], and the dashed blue line denotes the data fit with equation (viii). ELD_tot_ is calculated as equal to 5-HT_ads,eq_ at the maximum signal, and K_D_ is calculated as equal to [5-HT] at half the maximum signal, Figure S2, Fouling of Au-CNT electrodes in (a) 10 µM and (b) 1 mM 5-HT, showing CV cycles 1, 25, and 360. (t_acc_ = 0 min, scan rate = 50 mV/s). Insets: Ipa measured at each cycle. Percent of signal retained after 360 cycles is labeled. Rate of 5-HT fouling increases with increasing 5-HT concentration, Figure S3, (a) Effect of scan rate and t_acc_ on CV detection of 100 nM 5-HT. Error bars denote standard deviation (n = 2 electrodes). (b–d) Representative CV curves from (a) using a t_acc_ of 30 min and scan rate of (b) 50, (c) 200, and (d) 400 mV/s. 5-HT peak denoted with black arrow, Figure S4, Ipa measurement by subtraction of linear baseline fit (red dashed line). (a) PBS measurement, no CV peak. (b) 10 µM 5-HT Ipa measurement. Green brackets denote data points before the CV curve, selected for linear regression, which is extended to a point after the peak potential. The Ipa value is calculated by vertical subtraction of the background fit line from the CV curve, and selecting the maximum difference, Figure S5, Negative control CV measurements of AITC and ionomycin, showing no peak response, Figure S6, Selectivity of Au-CNT electrode in the presence of 100 µM uric acid (UA), 1 mM ascorbic acid (AA), 5 µM dopamine (DA), and 5 µM 5-HT in PBS. The black curve shows the combination of all chemicals. Both UA and AA show no current response within the working potential range. DA oxidation is measured as 3.2 µA at 0.23 V, while 5-HT oxidation is measured as 3.6 µA at 0.42 V, indicating significant separation of their peaks (t_acc_ = 10 min, n = 1). Figure S7, Electrode variability, measured as repeatability standard deviation (RSD%), over 50 cycles of fouling, evaluated from Figure 5b. RSD is calculated as standard deviation/mean × 100% (n = 4 electrodes).
