# Supplementary material for: Adsorption Kinetic Model Predicts and Improves Reliability of Electrochemical Serotonin Detection
Source: Methods Protoc. 2023 Jan 9;6(1):6. doi: 10.3390/mps6010006 (PMC9844352; doi:10.3390/mps6010006)
Supplement: Supplementary file 1 [file mps-06-00006-s001.zip › mps-2095487-supplementary.pdf]

methods and protocols

Supplementary

Adsorption Kinetic Model Predicts and Improves Reliability of Electrochemical Serotonin Detection

Ashley Augustiny Chapin, Jinjing Han and Reza Ghodssi

Derivation of Langmuir Isotherm Equation

This section describes the step-by-step derivation of the Langmuir isotherm equation that describes binding between 5-HT and the electrode (ELD). The terms here are specified for this reaction, but this process can be generalized for any binding reaction.

The 5-HT binding reaction with ELD states:

$$\text{5-HT} + \text{ELD} \leftrightarrow \text{5-HT}_{\text{ads}} \quad (i) \tag{1}$$

where 5-HT is the concentration of free 5-HT in solution (M) and ELD is the number of available binding sites on the electrode surface (#mol sites). The forward and reverse reactions rates are k_{on} (M^{-1} s^{-1}) and k_{off} (s^{-1}), respectively. The rate of production of 5-HT_{ads} and ELD binding sites can be described by the differential equations:

$$\frac{\text{d5-HT}_{\text{ads}}}{\text{dt}} = k_{on} \cdot [\text{5-HT}] \cdot \text{ELD} - k_{off} \cdot \text{5-HT}_{\text{ads}} \quad (ii) \tag{2}$$

$$\frac{\text{d}ELD}{\text{dt}} = k_{off} \cdot \text{5-HT}_{\text{ads}} - k_{on} \cdot [\text{5-HT}] \cdot \text{ELD} \quad (iii) \tag{3}$$

Derivation of the Langmuir isotherm requires the assumption that the binding reaction proceeds to equilibrium. At equilibrium, the rate of product formation = 0, such that:

$$k_{on} [5\text{-}HT] \cdot ELD_{eq} = k_{off} \cdot 5\text{-}HT_{ads,eq} \quad (iv) \tag{4}$$

where ELD_{eq} and 5-$HT_{ads,eq}$ are the number of available electrode binding sites (mol) and number of adsorbed 5-HT (mol) at equilibrium, respectively. The dissociation constant, K_D is described as:

$$K_D = \frac{k_{off}}{k_{on}} \; (v) \tag{5}$$

Plugging this into equation (iv), we obtain:

$$K_D = \frac{[5\text{-}HT] \cdot ELD_{eq}}{5\text{-}HT_{ads,eq}} \quad (vi) \tag{6}$$

ELD_{eq} can be expressed as:

$$ELD_{eq} = ELD_{tot} - 5\text{-}HT_{ads,eq} \quad (vii) \tag{7}$$

where ELD_{tot} is the total number of available binding sites on a fresh electrode, which can be experimentally estimated. Plugging (vii) into (vi) and solving for 5-$HT_{ads,eq}$ yields the Langmuir isotherm for this binding reaction:

$$5\text{-}HT_{ads,eq} = \frac{[5\text{-}HT] \cdot ELD_{tot}}{K_D + [5\text{-}HT]} \quad (viii) \tag{8}$$

It should be noted that this equation assumes excess [5-HT] in solution. This equation can then be used to experimentally determine K_D and ELD_{tot} for the tested electrodes. A

sample Langmuir isotherm is shown in Figure S1, which is fit to sample experimental data.

Experimental data acquisition

Here, data is acquired by electrochemically measuring 5-HT at increasing concentrations. When using CV, Ipa signal is obtained, which should scale with concentration up to a saturation point. Ipa is converted to 5-HT_{ads} by equation (6) in the main text, and because these measurements are each made at equilibrium (15 h allowed for binding to equilibrate per data point), we can label the y axis as 5-$HT_{ads,eq}$. This allows the data to be fit with the Langmuir isotherm equation (viii) to solve for the remaining variables.

Calculating K_D and ELD_{tot}

The plateau value is equal to ELD_{tot}, as the maximum number of binding sites for 5-HT on the electrode surface. K_D can be calculated as the [5-HT] at which the graph reaches half of the plateau value.

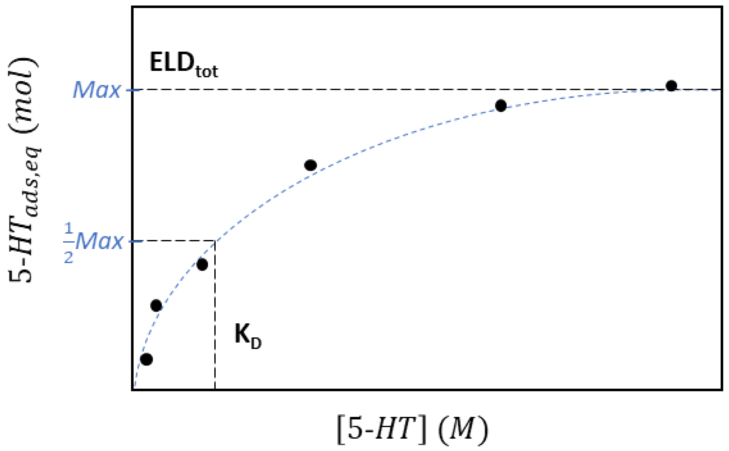

Figure S1. Sample Langmuir isotherm. Black dots show experimental data plotted as 5-$HT_{ads,eq}$ against [5-HT], and the dashed blue line denotes the data fit with equation (viii). ELD_{tot} is calculated as equal to 5-$HT_{ads,eq}$ at the maximum signal, and K_D is calculated as equal to [5-HT] at half the maximum signal.

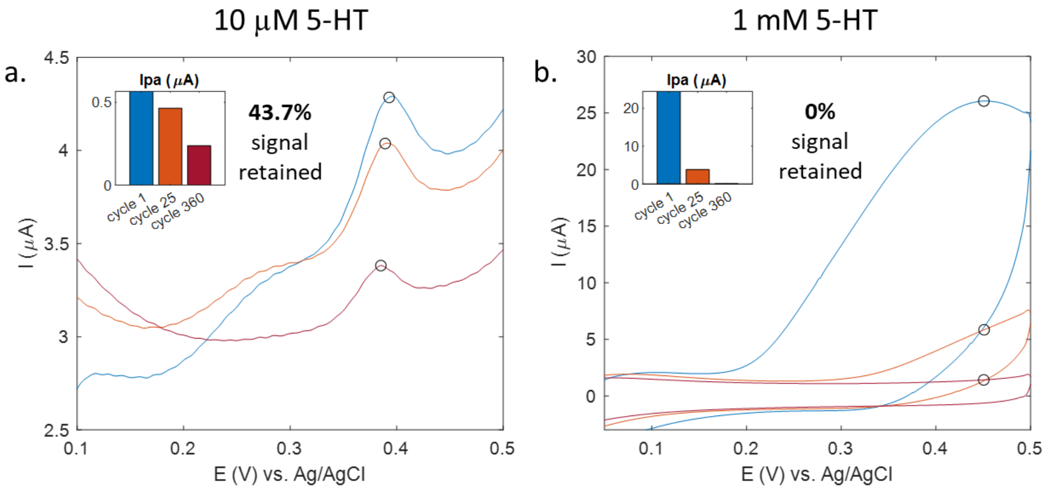

Figure S2. Fouling of Au-CNT electrodes in (a) 10 µM and (b) 1 mM 5-HT, showing CV cycles 1, 25, and 360. (t_{acc} = 0 min, scan rate = 50 mV/s). Insets: Ipa measured at each cycle. Percent of signal retained after 360 cycles is labeled. Rate of 5-HT fouling increases with increasing 5-HT concentration.

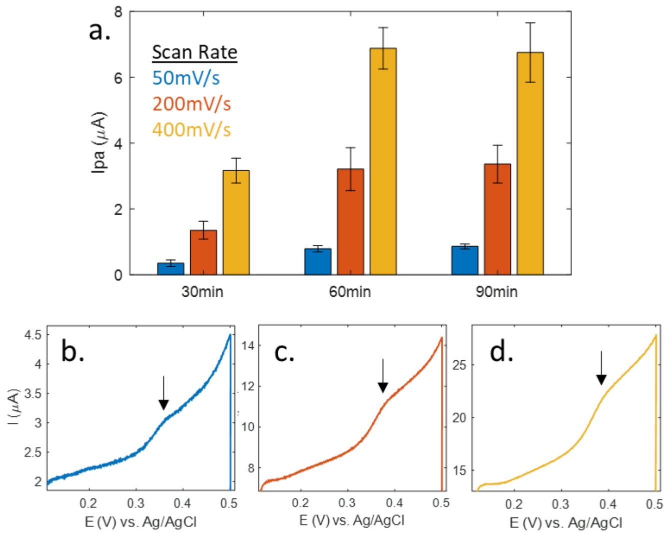

Figure S3. (a) Effect of scan rate and t_{acc} on CV detection of 100 nM 5-HT. Error bars denote standard deviation (n = 2 electrodes). (b-d) Representative CV curves from (a) using a t_{acc} of 30 min and scan rate of (b) 50, (c) 200, and (d) 400 mV/s. 5-HT peak denoted with black arrow.

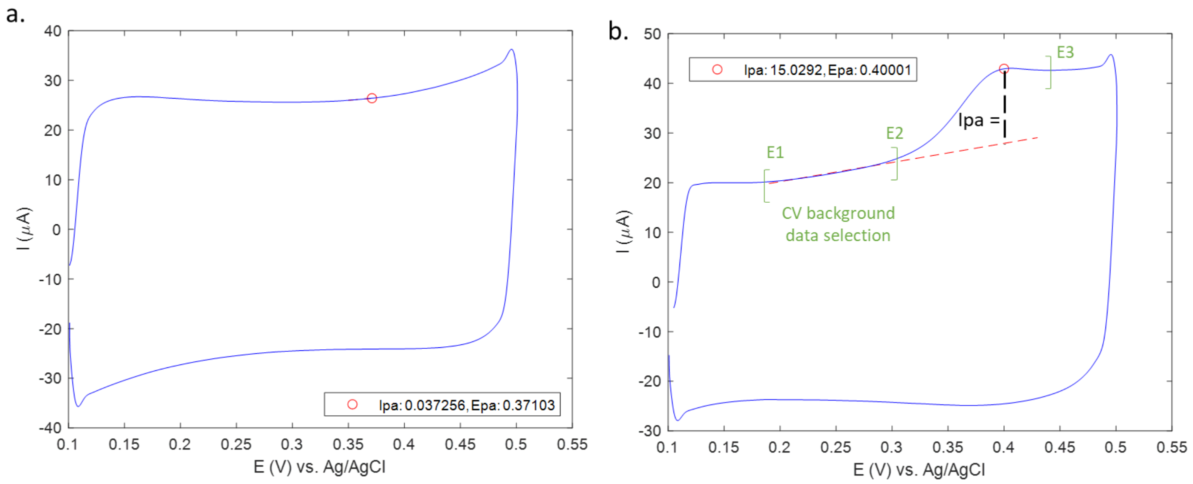

Figure S4. Ipa measurement by subtraction of linear baseline fit (red dashed line). **(a)** PBS measurement, no CV peak. **(b)** 10 μM 5-HT Ipa measurement. Green brackets denote data points before the CV curve, selected for linear regression, which is extended to a point after the peak potential. The Ipa value is calculated by vertical subtraction of the background fit line from the CV curve, and selecting the maximum difference.

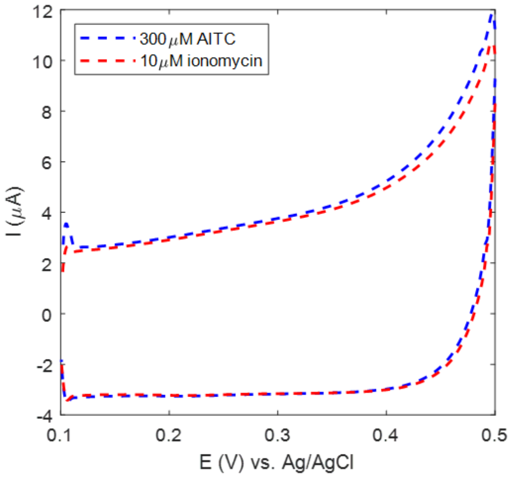

Figure S5. Negative control CV measurements of AITC and ionomycin, showing no peak response.

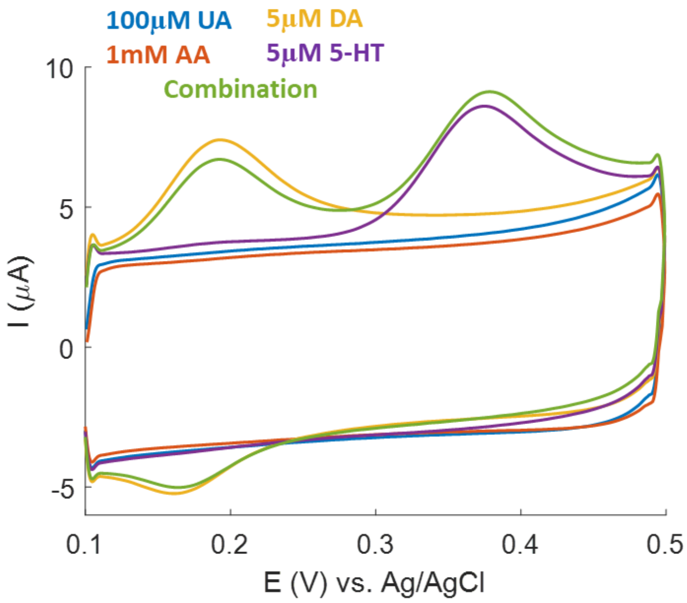

Figure S6. Selectivity of Au-CNT electrode in the presence of 100 μM uric acid (UA), 1 mM ascorbic acid (AA), 5 μM dopamine (DA), and 5 μM 5-HT in PBS. The black curve shows the combination of all chemicals. Both UA and AA show no current response within the working potential range. DA oxidation is measured as 3.2 μA at 0.23 V, while 5-HT oxidation is measured as 3.6 μA at 0.42 V, indicating significant separation of their peaks (t_{acc} = 10 min, n=1).

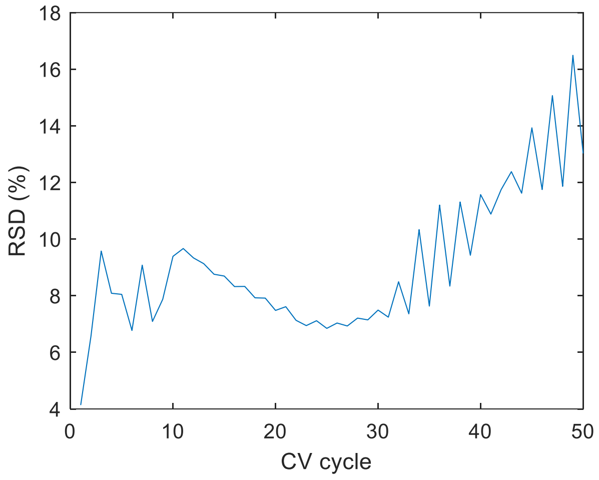

Figure S7. Electrode variability, measured as repeatability standard deviation (RSD%), over 50 cycles of fouling, evaluated from Figure 5b. RSD is calculated as standard deviation / mean x 100% (n = 4 electrodes).
